# Genetic counselling of young women with breast cancer for Li–Fraumeni syndrome: a nationwide survey on the experiences and attitudes of genetics professionals

**DOI:** 10.1007/s10689-018-0103-5

**Published:** 2018-09-20

**Authors:** J. J. Bakhuizen, M. E. Velthuizen, S. Stehouwer, E. M. Bleiker, M. G. Ausems

**Affiliations:** 1 Department of Genetics, University Medical Center Utrecht, PO Box 85090, 3508 AB Utrecht, The Netherlands; 2 Family Cancer Clinic, Netherlands Cancer Institute, Amsterdam, The Netherlands; 3 Division of Psychosocial Research and Epidemiology, The Netherlands Cancer Institute, Amsterdam, The Netherlands

**Keywords:** Attitudes, Li–Fraumeni syndrome, Genetics professional, Breast cancer, Genetic testing

## Abstract

Germline *TP53* mutations are associated with an increased risk of early-onset breast cancer. Traditionally, it was not standard practice to offer *TP53* genetic testing due to the low mutation detection rate and limited options regarding preventive screening. Recent guidelines recommend that all women diagnosed with breast cancer before the age of 31, irrespective of family history, should be offered *TP53* genetic testing. This study aims to gain more knowledge on the attitudes and experiences among genetics professionals regarding the timing and content of genetic counselling of young breast cancer patients for Li–Fraumeni syndrome (LFS). We conducted a nationwide online survey among genetics professionals who provide cancer genetic counselling in the Netherlands. Fifty-seven professionals completed the questionnaire (response rate overall 54%, clinical geneticists 70%). Most respondents reported that they discuss the option of *TP53* genetic testing—simultaneously with *BRCA 1*/*2*—during the initial counselling visit, especially in case of referral for treatment-focused genetic counselling. There was a general consensus about ten information items that should be discussed during counselling. Sixty-one percent of genetics professionals did not encounter difficulties in providing genetic counselling for LFS, but a substantial minority (29%) did. This study offers valuable insight, which will be useful for clinical practice. Studies which address young breast cancer patients’ attitudes and preferences regarding the timing and content of counselling are warranted to further determine the most appropriate genetic counselling strategy for these women.

## Introduction

Women in the Netherlands have a 12%, or a 1-in-8, lifetime risk of being diagnosed with breast cancer [1]. In 2017 almost 15,000 women have been diagnosed with breast cancer in the Netherlands [2]. The great majority (~ 80%) was older than 50 years of age at the time of diagnosis, but 93 women (0.6%) were younger than 30 years [2]. It is estimated that inherited mutations in breast cancer susceptibility genes account for 5–10% of all female breast cancers [3, 4]. Early age of onset is an indicator of genetic susceptibility [5]. In the Netherlands, all women diagnosed with breast cancer before the age of 40 are offered genetic testing for *BRCA1, BRCA2* and CHEK2* *1100delC* mutations [1, 6]. However, other cancer predisposition genes associated with early-onset breast cancer are known, including *TP53* associated with Li–Fraumeni syndrome (LFS) [7].

LFS is a rare, inherited cancer syndrome characterized by a very high risk of a wide variety of early-onset neoplasms, including sarcoma, breast cancer, brain tumours and adrenocortical carcinoma [8]. Breast cancer is the most common cancer among female mutation carriers. The peak of incidence is under 30 years [9]. Highest *TP53* mutation detection rates are reported in early-onset breast cancer patients with a family history suggestive of LFS or a personal history of an additional LFS-related tumour [10]. However, pathogenic TP53 sequence variants have also been described in women with apparently ‘sporadic’ early-onset breast cancer. Reported mutation detection rates in this population varied between 0 and 8.5% [11–16]. There was no consensus about offering *TP53* genetic testing to all early-onset breast cancer patients, irrespective of family history [10, 12, 13, 15, 16].

In the Netherlands, the general recommendation in 2005 and 2010 was to consider *TP53* germline mutation testing in women with breast cancer before the age of 30 [17, 18]. However, due to the limited preventive strategies, this testing was usually only offered to those with a family history suggestive of LFS. According to the latest national consensus-based guideline, *TP53* genetic testing should be offered to all breast cancer patients diagnosed before the age of 31 [19]. We hypothesized that the number of early-onset breast cancer patients counselled for LFS has increased over the last few years. However, little is known about genetics professionals’ experiences and attitudes towards the genetic counselling of young women with breast cancer for LFS in the absence of a suggestive family history. Their opinion is exceedingly important since they actually provide the counselling. Therefore, we conducted a survey among genetics professionals who provide cancer genetic counselling in the Netherlands. We investigated their views regarding the timing and content of *TP53* genetic counselling and the role of specialized professionals in psychosocial support. In addition, we collected nationwide laboratory records from all young breast cancer patients tested for *TP53* mutations in the Netherlands in order to assess the prevalence of *TP53* germline mutations. The results of this study are published separately [20]. In these two studies, we aimed to gain insight into the genetic counselling of young women with breast cancer for LFS in order to make clinical recommendations regarding the most appropriate counselling strategy for these women.

## Methods

### Study design and procedure

A cross-sectional online survey was conducted among all professionals (i.e. clinical geneticists, genetic counsellors and clinical geneticists-in-training) involved in cancer genetic counselling in the Netherlands. Study invitations with a link to the online survey were sent by mail. The initial time limit for completing the questionnaire was two weeks. Non-responders received up to two email reminders in an attempt to increase the response rate. Furthermore, key professionals (i.e. senior clinical geneticists from each hospital included in the study) were asked to encourage their colleagues to complete the survey. Data were collected in a 1 month period between March and April, 2017.

### Selection of participants

To identify potential respondents, email addresses of clinical geneticists and genetic counsellors with special expertise in cancer genetics were collected by using the member registration of the Dutch Cancer Genetics Group (WKO; a national working group of the Dutch Society of Clinical Genetics on cancer genetics) and the member registration of the Dutch Association of Genetic Counsellors (NVGC). Hospitals were contacted for missing data. Email addresses of clinical geneticists-in-training with some experience in oncogenetic counselling were gathered during an education meeting. In total, the contact details of 106 genetics professionals were collected, including 50 clinical geneticists, 32 genetic counsellors and 24 clinical geneticists-in-training.

### Questionnaire development

Questionnaire items were developed from unpublished results of a previous single-centre pilot study (Department of Genetics, University Medical Center Utrecht), including semi-structured interviews with genetics professionals, to identify key topics. A draft survey was designed using Research Online, a web-based secure and reliable survey tool that complies with the Good Clinical Practice guidelines for electronic data collecting [21]. The final questionnaire consisted of four sections. The first section comprised personal and professional demographics. The second section consisted of questions about the number of young breast cancer patients counselled for LFS by each respondent. Genetics professionals who have provided LFS genetic counselling for these counsellees were asked to fill in the third section of the questionnaire, including questions about providing genetic counselling for *BRCA 1*/*2-, CHEK2* and *TP53*-mutations during the initial counselling visit. Professionals without experience in this field could directly continue with the fourth section of the questionnaire. This final section assessed participants’ attitudes and views regarding the genetic counselling of young breast cancer patients for LFS. This included statements regarding information that could be provided for counselees (content of genetic counselling). Genetics professionals answered by providing a rating of importance to specific information. Other topics were the role of specialized professionals in psychosocial support and timing of *TP53* genetic counselling and testing. Questions were all closed-ended except for questions asking respondents to elaborate on their close-ended answers. Five-point Likert-type scales were used to rate the level of agreement (1 = strongly disagree, 2 = disagree, 3 = neutral, 4 = agree, 5 = strongly agree) and importance (1 = not at all important, 2 = of little importance, 3 = moderately important, 4 = important, 5 = very important) in the majority of items assessing attitudes and views. Other questions utilized pre-given categorical response options or dichotomous responses (“yes” or “no”).

### Data analysis

Statistical analyses were performed using the IBM Statistical Package for the Social Sciences (SPSS) 21.0. Descriptive statistics (percentages) were used for the analysis of all close-ended questions (quantitative data). For data analysis and interpretation of questions assessing genetics professionals’ attitudes, response categories for (agreement) Likert-type scale items were merged. Ratings 1 (strongly disagree) and 2 (disagree) were combined to construct a ‘(strongly) disagree’ category, and ratings 4 (agree) and 5 (strongly agree) were merged to construct a corresponding ‘(strongly) agree’ category. For interpretation of reported rates of importance regarding information items that could be discussed with counsellees, ratings 1 (very important) and 2 (important) were combined. In order to make recommendations for clinical practice, a threshold of 75% of respondents who rated the information item as ‘(very) important’ was considered to be ‘consensus’.

## Results

### Characteristics of respondents

Fifty-seven of 106 genetics professionals (54%) participated in the questionnaire (Table 1). The majority of respondents were clinical geneticists (61%) and more than half of respondents (53%) have worked in the field of oncogenetics for over 10 years. Response rates differed between professions and hospitals (data not shown) (“Appendix”).

**Table 1:** Characteristics of respondents (n = 57)

Variables and response categories	n (%)
Profession	
Clinical geneticist	35 (61%)
Genetic counsellor	13 (23%)
Clinical geneticist-in-training	9 (16%)
Experience (years) in oncogenetic counselling	
< 1	4 (7%)
1–2	8 (14%)
2–5	7 (12%)
5–10	8 (14%)
> 10	30 (53%)
Sex	
Male	9 (16%)
Female	48 (84%)

### Genetics professionals’ experiences

Ninety-six percent of respondents (55/57) have counselled at least one woman diagnosed with breast cancer before the age of 30 (Table 2). LFS was not discussed with all young breast cancer patients who were referred for cancer genetic counselling. Nonetheless, all professionals who have provided cancer genetic counselling for women with early-onset breast cancer have some experience in discussing LFS. Forty percent (23/57) of respondents have provided information about *TP53* genetic testing to at least ten young breast cancer patients. Genetics professionals were asked for reasons for not discussing LFS with all women who were counselled for early-onset breast cancer. The most reported reasons were changes in national or hospital-based guidelines. Discussing LFS with all young breast cancer patients has not always been part of standard care. Previously, genetic testing for *TP53* was only offered if a suggestive family history of LFS was present. Furthermore, two respondents preferred to wait for *BRCA* genetic test results before offering *TP53* genetic testing. *TP53* genetic testing is no longer indicated in case a *BRCA* mutation is detected.

**Table 2:** Genetics professionals’ experiences with the genetic counselling of women diagnosed with breast cancer under the age of 30 in general (I) and for LFS (II)

	Number of patients					
	0	1–5	5–10	10–15	15–20	> 20
I. Professionals who have counselled young breast cancer patients (n)	2	15	12	11	7	10
II. Professionals who have discussed LFS with young breast cancer patients (n)	–	20	11	10	5	8

*LFS* Li–Fraumeni syndrome

Seventy-three percent of professionals reported that, in general, they discuss the options for *BRCA1/2, CHEK2* and *TP53* during the initial counselling visit when women are referred for treatment-focused genetic counselling and testing (Table 3). Remaining respondents (27%) noted that they sometimes discuss these three genetic tests during the first appointment. In case of a regular referral, fewer counsellors (58%) provide genetic counselling for LFS during the initial visit.

**Table 3:** Genetics professionals’ experiences regarding providing genetic counselling for *BRCA 1/2, CHEK2* and *TP53*-mutations during the initial counselling visit

	n	Yes	Sometimes	No
Do you discuss the options of *BRCA1*/*2, CHEK2* and *TP53*-GT during the initial counselling visit?				
A. In case of referral for treatment-focused genetic counselling and testing^a^	49	73% (36)	27% (13)	–
B. In case of a regular referral	54	58% (33)	33% (19)	4% (2)

Evaluation	n	(Totally) disagree	Neutral	(Totally) agree
In general I am able to provide sufficient information about clinical and genetic aspects of BRCA1/2, CHEK2 and TP53-mutations during a single counselling visit	54	9% (5)	15% (8)	76% (41)
In general I am able to pay enough attention to the decision making process for BRCA1/2, CHEK2 and TP53-GT during a single counselling visit	54	15% (8)	24% (13)	61% (33)
I have difficulties in providing accurate genetic counselling for BRCA 1/2, CHEK2 and TP53 during a single counselling visit	51	55% (28)	18% (9)	28% (14)

Sample size (n) is presented and varies per question due to missing data and because of the fact that some questions do not apply to all respondents. Percentages reflect the proportion of participants selecting the response category divided by the total number of respondents to the corresponding question or statement

*LFS* Li–Fraumeni syndrome, *BC* breast cancer, *GT* genetic testing

^a^Referral for treatment-focused genetic counselling and testing is indicated when decisions about primary breast cancer treatment could be impacted by genetic test results

#### Evaluation

Respondents were asked to evaluate their experiences. The majority of counsellors (76%) (totally) agreed with the statement ‘In general I am able to provide sufficient information about clinical and genetic aspects of *BRCA1/2, CHEK2* and *TP53*-mutations during a single counselling visit.’ Fewer participants (61%) noted they were able to pay enough attention to the decision-making process. Approximately half of respondents (55%) did not have difficulties providing accurate genetic counselling for *BRCA1*/*2, CHEK2* and *TP53* during a single visit. Fourteen genetics professionals (28%; five clinical geneticists, four genetic counsellors and five clinical geneticists-in-training) experience difficulties in providing accurate genetic counselling for these four genes during a single visit.

### Genetics professionals’ attitudes

#### Content of genetic counselling

Genetics professionals rated the importance of 17 information items about LFS that could be discussed with young breast cancer patients (Table 4). Ten information items were rated as (very) important by more than 75% of participants. Of the category ‘tumour spectrum items’ all participants rated the following item as (very) important: ‘explaining that several cancers in addition to breast cancer may occur in individuals with LFS’. Fewer participants (35%) considered it important to mention the four most common tumour types in individuals with LFS. Providing information about the low *TP53* mutation detection rate, as well as the high risk of developing cancer in individuals with LFS, was rated as important by every professional. Mentioning specific odds (expressed as percentages) of mutation detection rates and cancer risks was considered (very) important by respectively 33 and 26% of respondents. With regard to other information items, almost all participants considered discussing possible consequences for family members, limited screening options as well as the option of additional support by a psychosocial professional as (very) important. Fewer participants (35%) considered mentioning the name of the genetic disorder to be important.

**Table 4:** Frequencies of genetics professionals’ ratings of information items as important or very important (n = 57)

During counselling, you should…	(Very) important	
	n	%
Tumour spectrum		
Explain that several cancers in addition to breast cancer may occur in individuals with LFS	57	100
Provide 1–3 examples of LFS-related cancers	46	81
Mention the four most common tumour types in individuals with LFS	25	44
Mutation detection rate		
Tell that the *TP53* mutation detection rate is (very) low	57	100
Specify the *TP53* mutation detection rate with a percentage	19	33
Cancer risks		
Explain that individuals with LFS are at high risk of developing cancer	57	100
Explain that individuals with LFS are at increased risk of developing a second cancer	45	79
Explain that individuals with LFS are at increased risk of developing multiple primary cancers	42	74
Specify cancer risk in individuals with LFS with a percentage	15	26
Tell that the overall lifetime cancer risk for women with LFS is higher than that for men	11	19
Other information items		
Discuss possible consequences for family members if a *TP53* mutation is detected	54	95
Mention there are limited screening options for *TP53* mutation carriers	53	93
Discuss the option of additional support by a psychosocial professional	51	90
Explain that radiotherapy should be avoided in individuals with LFS	49	86
Tell that *TP53* mutation carriers are offered a whole-body MRI (within research context)	47	83
Provide examples of possible reasons for or against performing *TP53* genetic testing	40	70
Mention the name of genetic disorder	20	35

*LFS* Li–Fraumeni syndrome, *MRI* magnetic resonance imaging

#### Additional psychosocial support

Genetics professionals were asked in which situation(s) additional support by a psychosocial professional (social worker or psychologist attached to the department of genetics) would be desirable. Respondents were allowed to choose more than one response category. The vast majority of respondents (88%) reported that additional support by a psychosocial professional is advisable in case it is questionable whether the counsellee understands the implications of *TP53* genetic testing (Table 5). Furthermore, doubts about performing *TP53* genetic testing, detection of a *TP53* mutation and problematic family communication were considered desirable indications for additional psychosocial support by more than two-thirds of respondents. In addition to the categorical response options provided, self-reported items included history of psychosocial disturbance/psychiatric disorder (n = 5) and patient’s request for additional psychosocial support (n = 5). Only two percent of respondents agreed that all young breast cancer patients who are offered *TP53* genetic testing should receive additional support by a psychosocial professional before *TP53* genetic testing is performed (Table 6, A).

**Table 5:** Genetics professionals’ opinions regarding additional psychosocial support (n = 57)

Additional support by a psychosocial professional^a^ is desirable…	n	%
In case it is questionable whether the counsellee has foreseen the implications of *TP53* GT	50	88
In case the counsellee is doubting about performing *TP53* GT	43	75
In case family communication is problematic	42	74
In case a *TP53* mutation is detected	39	68
In case a *TP53*-VUS is detected	18	32
In case of family history highly suggestive for LFS	10	18
For all counsellees who are offered *TP53* GT	3	5
For all women diagnosed with BC < 30 years	2	4
In case the counsellee wants to become pregnant	2	4

*BC* breast cancer, *GT* genetic testing, *LFS* Li–Fraumeni syndrome

^a^Social worker or psychologist

**Table 6:** Attitudes of genetics professionals regarding genetic counselling of women diagnosed with breast cancer < 30 years for LFS

	n	(Totally) disagree	Neutral	(Totally) agree
A. Additional psychosocial support				
All young BC patients who are offered diagnostic TP53 GT should receive additional support by a psychosocial professional before TP53 GT is performed	56	71%	27%	2%
B. Timing of genetic counselling and testing				
The option of TP53 GT should preferably be discussed after BRCA GT results are known	55	55%	24%	22%
In case of a regular referral, the option of TP53 GT should preferably be discussed after BRCA GT results are known	56	50%	21%	29%
In case of a regular referral, TP53 GT should preferably be performed after a second counselling visit	56	43%	25%	32%
C. In general				
I (would) encounter difficulties in discussing the option of BRCA GT and possible consequences of a BRCA mutation with women diagnosed with BC < 30 years	56	95%	2%	4%
I (would) encounter difficulties in discussing the option of TP53 GT and possible consequences of a TP53 mutation with women diagnosed with BC < 30 years	56	61%	11%	29%

Sample size (n) is presented and varies per question due to missing data. Percentages reflect the proportion of participants selecting the response category divided by the total number of respondents (per profession) to the corresponding statement

*LFS* Li–Fraumeni syndrome, *BC* breast cancer, *GT* genetic testing

#### Timing of genetic counselling and testing

Respondents were also asked about the extent of their agreement (or disagreement) with three statements about the timing of genetic counselling and testing for LFS (Table 6, B). The minority of respondents (22%) agreed with the first statement ‘The option of *TP53* genetic testing should preferably be discussed after *BRCA* genetic test results are known.’ A similar number of participants agreed with the second statement (‘In case of a regular referral…’). Less than one-third of respondents (32%) agreed that, in case of a regular referral, *TP53* genetic testing should preferably be performed after a second counselling visit. For all three statements, approximately one-fourth of respondents selected the ‘neutral’ response option.

#### In general

Almost all (96%) respondents (totally) disagreed with the statement ‘I (would) encounter difficulties in discussing the option of *BRCA1*/*2* with young breast cancer patients’. Only two clinical-geneticists-in-training agreed with this statement (Table 6, C). More respondents (29%) reported that they encounter difficulties in discussing the option of *TP53* genetic testing. Nonetheless the majority (61%) disagreed. Genetics professionals who reported that they encounter difficulties were asked to explain their responses. Table 7 shows the frequencies of reported reasons for encountering difficulties in providing genetic counselling of young breast cancer patients for LFS. Respondents were allowed to choose more than one response category. The most frequently noted reasons were ‘limited screening options’ (n = 25) and ‘severity and diversity of tumour spectrum’ (n = 21).

**Table 7:** Frequencies of reported reasons for encountering difficulties in providing genetic counselling of young breast cancer patients for LFS (n = 56)

	n	%
Limited screening options for *TP53* mutation carriers	25	45
Severity and diversity of tumour spectrum in *TP53* mutation carriers	21	38
The psychosocial distress that might be induced	12	21
Young age at diagnosis (before age 30)	5	9
Low *TP53* mutation detection rate	5	9
Little experience in providing genetic counselling for LFS	5	9

*LFS* Li–Fraumeni syndrome

## Discussion

To our knowledge, this is the first reported survey about the attitudes and experiences of genetics professionals regarding genetic counselling of young breast cancer patients for LFS. To summarize, most respondents reported that they discuss the option of *TP53* genetic testing—simultaneously with *BRCA 1/2*—during the initial counselling visit, especially in case of referral for treatment-focused genetic counselling. Furthermore, there was a general consensus about ten information items that should be discussed with counsellees. Almost two-thirds of genetics professionals did not encounter difficulties in providing genetic counselling of young breast cancer patients for LFS. However, a substantial minority did.

### Clinical recommendations

This study aimed to make recommendations for clinical practice regarding the content and timing of genetic counselling and indications for additional psychosocial support. Table 8 shows the ten information items about LFS that should be discussed with young breast cancer patients during pre-test counselling.

**Table 8:** Information items about LFS that should be discussed during pre-test genetic counselling

Several cancers in addition to breast cancer may occur in individuals with LFS
1–3 examples of LFS-related cancers
The *TP53* mutation detection rate is (very) low
Individuals with LFS are at high risk of developing cancer
Individuals with LFS are at increased risk of developing a second cancer
Possible consequences for family members if a *TP53* mutation is detected
There are limited screening options for *TP53* mutation carriers
The option of additional support by a psychosocial professional
Radiotherapy should be avoided in individuals with LFS
*TP53* mutation carriers are offered a whole-body MRI (within research context)

*LFS* Li–Fraumeni syndrome, *MRI* magnetic resonance imaging

Almost all respondents indicated that it is important to discuss the option of additional support by a psychosocial professional during genetic counselling. However, the majority of genetics professionals disagree that all young breast cancer patients who are offered diagnostic *TP53* genetic testing should receive this support before *TP53* genetic testing is performed. These findings suggest that genetics professionals feel capable of identifying counsellees who need additional psychosocial support. Nonetheless, there seems to be general consensus among respondents (≥ 75%) that additional support by a psychosocial professional should be provided in two specific situations. Firstly, in case it is questionable whether the counsellee has understood the implications of *TP53* genetic testing, and secondly, in case the counsellee has doubts about performing *TP53* genetic testing. Our results partly differ from recommendations from the Dutch patient association ‘Stichting Diagnose Kanker’ (SDK) and European Li–Fraumeni Families Foundation (ELFF), as mentioned in the medical standard ‘Li–Fraumeni Syndroom’. A substantial minority of respondents (approximately one-third) did not consider ‘the detection of a *TP53* mutation’ as an indication for specialized psychosocial support. Whereas, the medical standard recommends that specialized psychosocial counselling should always be provided in case a *TP53* mutation is detected [22]. A plausible explanation for this discrepancy is that the majority of respondents were clinical geneticists who have been working in the field of oncogenetics for over 10 years.

### Difficulties in providing genetic counselling for LFS

The results of our retrospective laboratory records review showed an increase in the number of early-onset breast cancer patients tested for *TP53* germline mutations [20]. This suggests that *TP53* genetic testing is being discussed with young breast cancer patients more often. Although, most respondents did not encounter difficulties in providing genetic counselling of young breast cancer patients for LFS, a substantial minority did. The most frequently noted reasons were limited screening options, severity and diversity of tumour spectrum and the psychosocial distress that might be induced.

In recent years, multiple suggestions for clinical surveillance of individuals with LFS have been proposed [7, 23–26]. In the Netherlands, *TP53* mutations carriers are offered an annual surveillance program, including physical examination, blood tests, whole body MRI (WB-MRI) and breast MRI in female patients. Recently, the initial findings of this surveillance program were published [27]. Malignancies were detected in approximately 7% of individuals with LFS. This detection rate must be weighed against the limitations (e.g. many false-positive findings and additional diagnostic procedures). Furthermore, it is uncertain whether annual WB-MRI will improve the long-term prognosis of *TP53* mutation carriers. Data on *TP53* carriers’ experiences with the annual surveillance program, including the psychosocial issues, are currently being collected.

A few studies have reported that a substantial proportion of individuals in families with *TP53* mutations, irrespective of their carrier status, exhibit psychological distress [28–30]. However, to our knowledge, no studies have been published evaluating the psychosocial impact of discussing LFS with early-onset breast cancer patients. As ‘sporadic’ early-onset breast cancer patients lack a family history suggestive of LFS, they may experience different psychosocial issues. Studies which address young breast cancer patients’ experiences regarding the genetic counselling for LFS are warranted, in order to gain insight into the psychosocial impact of this counselling and to help identify those individuals in need of professional psychosocial support.

### Limitations

Although a large number of genetics professionals did participate in the questionnaire, the results of the survey might not be generalizable for all genetics professionals who provide cancer genetic counselling in the Netherlands. Forty-eight percent of eligible genetics professionals did not participate in the questionnaire and response rates differed between professions and hospitals. A detailed stratified analysis on profession was not possible due to the significant difference between the number of participants in each profession. Additionally, the possibility of a non-response bias cannot be excluded, as it is possible that professionals who have responded have stronger opinions than non-respondents. However, all nine hospitals were represented in the group of respondents and this study covered all three professions that provide genetic counselling. The response rate among clinical geneticists (supervisors of genetic counsellors and genetics residents) was relatively high (70%) and exceeded the mean response rate of 56% reported in a review of postal surveys conducted among healthcare professionals [31]. Furthermore, more than half of the respondents have extensive experience in providing cancer genetic counselling as they have been working in cancer genetic counselling for over 10 years.

## Conclusion

The number of early-onset breast cancer patients tested for LFS has increased over the past years.

The results of our nationwide survey suggest that most genetics professionals discuss the option of *TP53* genetic testing—simultaneously with *BRCA 1*/*2* and *CHEK2*—during the initial counselling visit, especially in case of referral for treatment-focused genetic counselling. Our recommendations regarding the content of genetic counselling for LFS and indications for additional psychosocial support will be useful for clinical practice. Studies which address young breast cancer patients’ experiences (including psychosocial impact) are needed to further determine the most appropriate genetic counselling strategy for these women.

## Appendix

See Table 9.

**Table 9:** Response rates per profession and hospital

	Invitations (n)	Respondents (n)	Response rate (%)
Profession			
Clinical geneticist	50	35	70
Genetic counsellor	32	13	41
Clinical geneticist-in-training	24	9	38
Hospital			
AMC	8	3	38
AVL	10	8	80
Erasmus MC	12	4	33
LUMC	14	8	57
MUMC	11	4	36
Radboudumc	17	9	53
UMCG	14	7	50
UMCU	12	9	75
VUMC	9	5	56
Total	106	57	54
